# Supplementary material for: Effectiveness and Safety of Erector Spinae Plane Block vs. Conventional Pain Treatment Strategies in Thoracic Surgery
Source: J Clin Med. 2025 Apr 22;14(9):2870. doi: 10.3390/jcm14092870 (PMC12072521; doi:10.3390/jcm14092870)
Supplement: Supplementary file 1 [file jcm-14-02870-s001.zip › jcm-3535796-supplementary.pdf]

Table S1. Patient Demographics and Characteristics of patients that stayed in PACU for longer than 6 hours

	VATS patients (N = 25)			thoracotomy patients (N = 25)		
	with ESPB (N = 18)	with IV-CA (N = 7)	*P*	with ESPB (N = 12)	with TEA (N = 13)	*P*
age, years	58.3 (22.4)	58.6 (20.1)	0.98	61.2 (10.2)	62.2 (11.2)	0.81
sex, female	8 (44.5)	3 (42.9)	0.94	4 (33.3)	4 (30.8)	0.89
BMI, mean (SD)	26.5 (6.4)	23.2 (3.3)	0.13	28.9 (7.3)	25.0 (3.5)	0.11
ASA score	3.0 [2.8 to 3.0]	3.0 [2.8 to 3.0]	0.37	3.0 [2.0 to 3.0]	3.0 [3.0 to 3.0]	0.47
type of surgery			0.80			N.A.
elective surgery, n (%)	15 (83.3)	6 (85.7)		12 (100.0)	13 (100.0)	
urgent surgery, n (%)	3 (16.7)	1 (14.3)		0 (0.0)	0 (0.0)	
analgesia						
ESPB, n (%)	18 (100.0)	N.A.		12 (100.0)	N.A.	
before surgery	2 (11.1)	N.A.		0 (0.0)	N.A.	
TEA	0 (0.0)	0 (0.0)		0 (0.0)	13 (100.0)	
before surgery	N.A.	N.A.		N.A.	13 (100.0)	
primary diagnosis			0.21			0.21
malignancy	11 (61.1)	2 (28.6)		9 (75.0)	10 (76.9)	
pathology pending	5	1		0	2	
adenocarcinoma	2	0		2	2	
squamous cell carcinoma	2	1		4	2	
non–small cell lung cancer	1	0		1	0	
metastasis	0	0		0	0	
other malignancy	1	0		2	4	
pneumothorax	6 (33.3)	1 (14.3)		0 (0.0)	0 (0.0)	
pleural effusion	0 (0.0)	0 (0.0)		0 (0.0)	0 (0.0)	
haemothorax	0 (0.0)	1 (14.3)		0 (0.0)	0 (0.0)	
empyema	1 (5.6)	1 (14.3)		3 (25.0)	0 (0.0)	
emphysema	0 (0.0)	0 (0.0)		0 (0.0)	0 (0.0)	
pectus deformity	0 (0.0)	0 (0.0)		0 (0.0)	0 (0.0)	
other	0 (0.0)	2 (28.6)		0 (0.0)	3 (23.1)	

Data are presented as medians with interquartile ranges [IQR]; VAS used a scale of 1 to 10. Abbreviations: VAS, visual analogue scale. PACU, Post–anesthesia Care Unit; VATS, video assisted thoracic surgery; ESPB, erector spinae plane block; IV-CA, intravenous combination analgesia; TEA, thoracic epidural analgesia.

Table S2. Arterial Blood Gas Analysis Results in PACU

	VATS patients (N = 165)			thoracotomy patients (N = 72)		
	with ESPB (N = 101)	with IV–CA (N = 64)	*P*	with ESPB (N = 41)	with TEA (N = 31)	*P*
patients with arterial BGA	26 (25.7)	13 (20.3)	0.42	22 (53.7)	17 (54.8)	
PaO_2, kPa, median [IQR]	75.5 [68.7 to 88.0]	75.4 [61.9 to 94.2]	0.43	75.1 [63.2 to 101.8]	95.1 [84.4 to 152.0]	0.02
O_2 L/min, median [IQR]	2.0 [0.0 to 4.0]	2 [0.0 to 2.0]	0.31	2.0 [2.0 to 4.0]	2.0 [0.0 to 4.5]	0.32
$PaCO_2$, kPa, median [IQR]	45.3 [38.9 to 49.1]	47.1 [43.5 to 54.6]	0.08	43.1 [39.2 to 49.9]	43.3 [39.8 to 48.0]	0.97

Abbreviations: PACU, Post–anesthesia Care Unit; VATS, video assisted thoracic surgery; ESPB, erector spinae plane block; IV–CA, intravenous combination analgesia; TEA, thoracic epidural anesthesia; BGA, blood gas analysis; PaO_2, arterial partial pressure of oxygen; $PaCO_2$, arterial partial pressure of carbon dioxide; O_2 (l/min), supplemental oxygen via Venturi mask.

Table S3. Cost comparison.

	VATS patients (N = 165)		thoracotomy patients (N = 72)	
	with ESPB (N = 101)	with IV–CA (N = 64)	with ESPB (N = 41)	with TEA (N = 31)
costs per block or TEA (€)				
single use material	17.9	0	17.9	21.6
medication	51.7	0	51.7	25.6
total	69.6	0	69.6	47.2

Abbreviations: VATS, video assisted thoracic surgery; ESPB, erector spinae plane block; IV–CA, intravenous combination analgesia; TEA, thoracic epidural anesthesia.

Table S3. Cost comparison

This comparison includes cost for material and medication per single shot ESPB or TEA lasting at least 24 hours. It does not include costs for opioids and non-opioids, personnel costs, nor costs for non-single use medical equipment as syringe pumps or ultrasound machines.
